# Programmatic evaluation of feasibility and efficiency of at birth and 6-week, point of care HIV testing in Kenyan infant

**DOI:** 10.1371/journal.pone.0240621

**Published:** 2020-10-09

**Authors:** Catherine Wexler, Niaman Nazir, May Maloba, Melinda Brown, Kathy Goggin, Brad Gautney, Nicodemus Maosa, Shadrack Babu, Elizabeth Muchoki, Natabhona Mabachi, Raphael Lwembe, Sarah Finocchario-Kessler

**Affiliations:** 1 Department of Family Medicine, University of Kansas Medical Center, Kansas City, KS, United States of America; 2 Department of Preventive Medicine, University of Kansas Medical Center, Kansas City, KS, United States of America; 3 Global Health Innovations–Kenya, Nairobi, Kenya; 4 Children’s Mercy Kansas City, Health Services and Outcomes Research, Kansas City, MO, United States of America; 5 School of Medicine, University of Missouri-Kansas City, Kansas City, MO, United States of America; 6 Global Health Innovations, Dallas, TX, United States of America; 7 Kenya Medical Research Institute, Nairobi, Kenya; University of North Carolina at Chapel Hill, UNITED STATES

## Abstract

**Background:**

Testing infants at birth and with more efficient point of care (POC) HIV diagnostic can streamline EID and expedite infant ART initiation. We evaluated the implementation of at birth and 6-week POC testing to assess the effectiveness and feasibility when implemented by existing hospital staff in Kenya.

**Methods:**

Four government hospitals were randomly assigned to receive a GeneXpert HIV-1 Qual (n = 2) or Alere m-PIMA (n = 2) machine for POC testing. All HIV-exposed infants enrolled were eligible to receive POC testing at birth and 6-weeks of age. The primary outcome was repeat POC testing, defined as testing both at birth and 6-weeks of age. Secondary outcomes included predictors of repeat POC testing, POC efficiency (turnaround times of key services), and operations (failed POC results, missed opportunities).

**Results:**

Of 626 enrolled infants, 309 (49.4%) received repeat POC testing, 115 (18.4%) were lost to follow up after an at-birth test, 120 (19.2%) received POC testing at 6-weeks only, 80 (12.8%) received no POC testing, and 2 (0.3%) received delayed POC testing (>12 weeks of age). Three (0.4%) were identified as HIV-positive. Of the total 853 POC tests run at birth (n = 424) or 6-weeks (n = 429), 806 (94.5%) had a valid result documented and 792 (98.3%) results had documented maternal notification. Mean time from sample collection to notification was 1.08 days, with 751 (94.8%) notifications on the same day as sample collection. Machine error rates at birth and 6-weeks were 8.5% and 2.5%, respectively. A total of 198 infants presented for care (48 at birth; 150 at 6-weeks) without receiving a POC test, representing missed opportunities for testing.

**Discussion:**

At birth POC testing can streamline infant HIV diagnosis, expedite ART initiation and can be implemented by existing hospital staff. However, maternal disengagement and missed opportunities for testing must be addressed to realize the full benefits of at birth POC testing.

## Introduction

Although 76% of pregnant women living with HIV in Kenya have access to antiretroviral therapy (ART) to prevent mother-to-child transmission of HIV, 6,800 children still acquired HIV in 2019 [1]. Without timely treatment 50% of these children will die by 2 years of age [2]. Early infant diagnosis of HIV [EID] services are critical to identify infants living with HIV and initiate them on ART; however, only 69% of HIV-exposed infants in Kenya receive a virologic test [1]. Of those who are tested and identified as HIV-positive, 82% are initiated on treatment [1] at a median age of 17.1–25.1 weeks [3, 4], well beyond the target of 12 weeks to reduce the risk of mortality and slow disease progression [5].

A significant challenge to early diagnosis and treatment are the logistic barriers posed by virologic tests required for infants. In Kenya, PCR testing for EID is only available at central laboratories. This requires clinicians to collect dried blood spots from infants at the health facility, label and ship the samples via courier to the laboratory, wait for the laboratory to process the sample and post the result, and then recall the mother to the hospital for result notification. This multistep process can take 3.6–8 weeks from sample collection to caregiver notification [4, 6], and creates multiple opportunities for sample or result mismanagement and patient disengagement, thus preventing mothers from receiving their infant’s result and initiating treatment (if positive) [7, 8]. With recent guidelines recommending at birth and more frequent HIV testing for HIV-exposed infants in Kenya (2016) [9] and more frequent viral load tests for all patients on ART (2014) [10], the annual number of EID tests rose from 55,000 in 2015 to 140,000 by 2019 [11]. The demand for viral load testing has already evidenced a drastic increase from 237,000 in 2014 to 1.2 million in 2018 [12]. Meeting this increased demand is expected to strain the capacity of central laboratories [13] and, as a result, may further increase turnaround times for EID [14].

Testing infants earlier (at birth) and with a more efficient point of care (POC) HIV diagnostic technology is emerging as a strategy to streamline EID, minimize challenges with traditional central laboratory-based PCR testing, and bridge the gap between EID demand and laboratory capacity. POC diagnostic technologies such as GeneXpert HIV-1 Qual [15] and Alere m-PIMA [16] are cartridge-based tests that can be processed at the hospital by trained clinical or laboratory staff and can result in more rapid turnaround times of results, more infants being identified as HIV-positive and more infants initiated on ART at younger ages than traditional testing strategies [17–22]. Studies have shown high sensitivity and specificity of POC testing and found that POC implementation is feasible in hospital-based settings in Kenya [23, 24], South Africa [18, 25–27], Mozambique [28], and Tanzania [29]; is acceptable to providers [26] and patients [30]; and may be a cost-effective option for EID [31, 32]. Based on the promising evidence supporting POC, the World Health Organization (WHO) conditionally recommended the introduction of POC for EID in 2016 [33]. Kenya and other countries have begun incorporating at birth and POC testing into national plans for EID [34–36].

Despite this promising evidence, questions remain regarding the implementation and impact of at birth POC testing. Key findings suggest lower sensitivity and higher assay error rates of POC among newborns compared to older infants and children [18, 25, 26], reduced likelihood of repeat HIV testing at 6–10 weeks of age among infants tested at birth [19], provider reluctance to use POC results for treatment initiation [23], and the need to add additional staff to support at birth POC testing [18]. In order to answer these outstanding questions, the objectives of this evaluation were to assess: (1) feasibility of at birth POC testing, (2) early retention in POC testing at birth and 6-weeks, (3) efficiency of POC testing (turn-around time), and (4) operational challenges using two different POC platforms for infant HIV testing at birth and at 6-weeks of age at four government hospitals in Kenya when implemented by existing clinical personnel in hospital-based settings in Kenya.

## Methods

### Overview and setting

We conducted an evaluation assessing the feasibility of implementing two emerging strategies for infant HIV testing in Kenyan hospitals: point of care testing at birth and at 6-weeks of age. Medium to high volume, government-funded hospitals (8–15 EID patients per month) in Kisumu County (2 hospitals), Nakuru County (1 hospital), and Mombasa County (1 hospital) were eligible for inclusion. These counties were selected because they are high burden areas where our team had established study infrastructure, including strong working relationships with the county health department and existing research assistants in the area. The four study hospitals were randomly assigned to receive a GeneXpert HIV-1 Qual (GeneX, n = 2) or Alere m-PMIA (n = 2) for POC testing. A detailed description of procedures has been previously published [37].

### Participant eligibility

All pregnant women living with HIV who were >18 years of age and presented for antenatal/PMTCT, maternity, or EID care prior to 24 weeks of infant age at the implementing hospital from June 2017 to November 2018 were eligible to enroll. All eligible candidates were informed about the purpose of the research, potential benefits and risks, and the procedures by trained research or clinical staff. Women who enrolled during antenatal/PMTCT care were counseled on at birth testing and advised to return to the implementing hospital for delivery or by 2 weeks of infant age.

### Procedures

All infants of mothers living with HIV enrolled were eligible to receive POC testing using their hospital’s designated POC machine at birth and at 6-weeks of age. These time-points aligned with PCR testing time points outlined in the 2016 Kenyan National Guidelines for EID [9]; though at the time of the implementation at birth testing was not routinely implemented as national piloting had not occurred. At birth sample collection occurred in the maternity department prior to maternal/infant discharge (for infants born at the study hospital). For infants who presented for a birth test after delivery and for 6-week testing, samples were collected in the maternal and child health department (MCH). All samples were whole blood collected via heel-stick and were loaded into the POC machine’s disposable cartridge and then carried to the machine, which was in either the maternity (1 hospital), MCH (2 hospitals), or hospital’s on-site laboratory (1 hospital), based on layout and available space at each of the hospitals. All POC sample collection and processing was conducted by on-site healthcare providers. Sample processing took approximately 90 minutes (GeneX) [38] or 52 minutes (Alere m-PIMA) [39]. Mothers were advised to wait for the sample to be processed for same-day notification of results; however, a few were unable to and they were notified at a later visit. At each time point, an additional PCR sample was collected and processed per standard of care procedures: a dried blood spot card was sent by courier to the hospital’s designated central laboratory, processed, and then results were posted in the national database, which could be accessed by the facility. At both time points, if positive by POC or PCR, the infant was linked to the comprehensive care center (CCC) for ART initiation. If negative, mothers were counseled on re-testing recommendations.

#### Staffing and responsibilities

Prior to implementation, a 2-day training was provided to clinical personnel from relevant hospital departments (antenatal care, maternal and child health, maternity, laboratory, CCC). Training covered protocols (day 1) and use of their hospital’s designated POC machine (day 2). Part-time site coordinators, who made periodic site visits to implementing hospitals, supported research-specific tasks (data entry and cleaning), patient follow up, and contacting POC manufacturers regarding errors or machine breakdown at each site. Existing clinical staff were primarily responsible for conducting at birth POC testing, including: counseling patients throughout antenatal care on at birth POC testing; conducting informed consent; and sample collection, processing, and result notification at both time points; and treatment initiation if applicable.

### Measures

The primary outcome was the proportion of infants with repeat POC testing through 6-weeks of age. Testing time points and retention categories are defined in Table 1.

**Table 1:** Definitions of evaluation measures.

Term	Definition
Testing Time-points	
At birth test	Sample collected at 0–4 weeks of age
On-time birth test	Sample collected 0–2 weeks of age
6-week test	Sample collected at 4–12 weeks of age
On-time 6w test	Sample collected at 4–8 weeks of age
Retention Definitions	
Repeat POC testing	Receipt of both at birth and 6-week POC
Loss to follow up	Receipt of at birth POC, but not 6-week POC
Standard POC^a^	Receipt of 6-week POC, but not at birth POC
No POC	Enrolled <12 weeks of age but did not receive an at birth or 6-week POC
Delayed EID	Did not enroll until >12 weeks
Incomplete POC	Combined: Lost to follow up, Standard POC, No POC, delayed EID
Missed opportunity	Infant presented for care at given time-point but no POC test was conducted (e.g. due to machine or provider errors, cartridge stock outs, etc)

^a^Equivalent to testing time points for routine EID services at the time of implementation, as national piloting of at birth testing occurred.

The proportions receiving at birth tests by 2 weeks and 6-week tests between 4–8 weeks, i.e. “on time” testing, were calculated. We also calculated at birth and 6-week testing using broader definitions of 0–4 weeks and 4–12 weeks, respectively. This decision was made for two reasons: 1) with typical definitions, testing between 2–4 weeks would not be included as either an at birth or a 6-week test and, thus, would underestimate the proportion of infants receiving early testing and 2) while 4–8 weeks is the typical definition for 6-week PCR testing with longer turnaround time, we expanded this to 12 weeks for POC since–hypothetically–testing infants with POC up to 12 weeks of age would allow for ART initiation by the targeted 12 weeks of age. Infants who presented for care outside of these designated windows were tested upon presentation for care; however, their testing data was ineligible for inclusion in this analysis.

Secondary outcomes included predictors of repeat POC testing, POC efficiency (age at infant testing, turnaround times of key services [sample processing, mother notification of result, ART initiation] and operational challenges (proportion of failed POC results and numbers of and reasons for missed opportunities).

### Analyses

A national nurse’s strike from June 6, 2017 to November 3, 2017 nearly halted ANC, delivery, and EID services at hospitals throughout the country; thus, to eliminate the effect of the strike on retention outcomes, we limited our analyses to infants born between November 3, 2017 and November 3, 2018. We also included mother-infant pairs enrolled antenatally with an estimated delivery date between this window, but no documented infant DOB (i.e., pair was loss to follow up prior to infant birth). We excluded mother-infant pairs who were discharged due to documented transfer/move to another facility, infant mortality, maternal mortality, miscarriage/stillbirth, or withdrawn consent.

We assessed the proportion of infants eligible for each test based on timing of enrollment and the proportion who fell into each of the retention categories. We assessed bivariable predictors of repeat POC testing, by comparing infants who received repeat POC testing with those who received incomplete POC testing. Independent variables (described in Table 2) were analyzed using the Chi-square test and non-parametric Fisher’s exact test when the expected number of values in one or more cells was less than 5 for dichotomous variables or using non-parametric Wilcoxon rank sum test for continuous variables. To assess POC efficiency, we calculated the proportion of POC samples with same day results and mean turnaround times for each key step for POC testing (sample collection to mother notification, mother notification to ART initiation). To assess POC testing operational challenges, we assessed the proportion of POC samples that resulted in processing errors and missed opportunities and then described reasons for missed opportunities.

**Table 2:** Characteristics of participants.

	N^a^	%
Maternal age (median, IQR)	30.35	25.5–34.0
Hospital	624	
Hospital 1	162	26.0%
Hospital 2	195	31.3%
Hospital 3	163	26.1%
Hospital 4	104	16.7%
Timing of enrollment	626	
Antenatally	457	73.0%
Labor/delivery	55	8.8%
Postnatally	114	18.2%
Maternal education	412	
No formal education	21	5.1%
Some primary	235	57.0%
Some secondary	127	30.8%
University/ college	29	7.0%
Timing of maternal ART	524	
Newly initiated	126	24.0%
Previously initiated	416	79.4%
Maternal marital status	512	
Married/living with partner	453	88.5%
Separated/not living with partner	59	11.5%
Travel time to hospital	496	
<60 minutes	411	82.9%
>60 minutes	85	17.1%
Infant sex	617	
Female	302	48.9%
Male	315	51.1%

^a^Total sample size differs between variables due to missing data.

## Ethical statement

All participants provided written informed consent prior to enrollment. The protocol was approved by the Institutional Review Boards at the Kenya Medical Research Institute (SSC 3390) and the University of Kansas Medical Center (Study 00140399).

## Results

A total of 1,000 mother-infant pairs were enrolled. We excluded a total of 374 mother-infant pairs, including: 330 pairs with an infant DOB outside of the analysis period, 11 pairs with no documented maternal date of birth, 2 pairs with maternal age <18 years at enrollment, and 31 pairs who were discharged early due to transfer/move to another facility (n = 11), infant mortality (n = 14), maternal mortality (n = 1), miscarriage/stillbirth (n = 4), or withdrew consent (1). Thus, a total of 626 infants with a DOB or expected delivery date between November 3, 2017 and November 3, 2018 were included in final analyses. Descriptive statistics for the sample are shown in in Table 2.

### Retention

Of the 626 infants included in analysis, 590 (94.2%) were enrolled either antenatally or within four weeks of infant birth and thus eligible for both POC tests, 34 (5.4%) were enrolled when the infant was between ≥ 4 and 12 weeks of age and, thus, were only eligible for the 6-week POC test, and 2 (0.3%) were enrolled at >12 weeks of age and, thus, were not eligible for any of the targeted tests.

**Fig 1:**
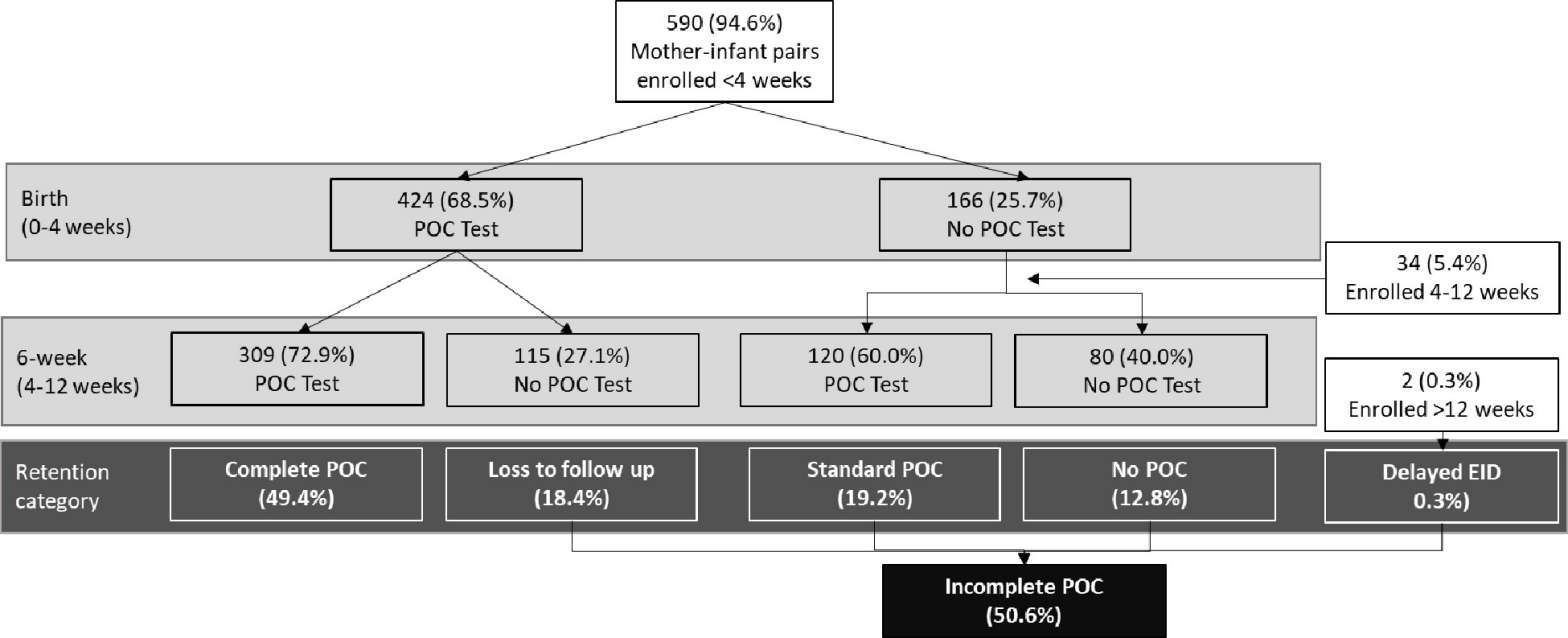
Repeat POC testing at birth and 6-weeks.

Of all enrolled infants, a total of 544 (86.9%) had at least one POC sample collected by 12 weeks of age, with 309 (49.4%) receiving both an at birth and a 6-week POC test, achieving repeat POC testing through 6-weeks. A total of 317 (50.6%) infants received incomplete POC testing: 115 (18.4%) were lost to follow up, 120 (19.2%) received only standard POC testing at 6-weeks, 80 (12.8%) received no POC test, and 2 (0.3%) received delayed EID. Infants who received a POC test at birth were significantly more likely to receive a POC test at 6-weeks compared to infants who did not receive a POC test at birth: 309 of the 424 (72.9%) infants who received an at birth POC test also received a 6-week POC test, compared to 120 of the 202 infants who did not receive an at birth POC test (59.4%, p<0.01). Timing of enrollment and receipt of POC testing is detailed in Fig 1.

Of the 424 infants receiving an at birth POC test, 343 (80.9%) received it on-time and 81 (19.1%) received it late. Of the 429 infants receiving a 6-week POC test, 412 (96.0%) received it on time and 17 (4.0%) received it late. In total, 257 (41.1%) infants received both at birth and 6-week POC tests on time.

In bivariable analyses, complete POC testing varied significantly by hospital of enrollment and timing of enrollment. Rates of complete POC were 35.9%, 54.0%, 55.8% and 56.8% at the four hospitals (p<0.0001). The proportion of mother-infant pairs achieving complete POC testing also varied by timing of enrollment with similar rates among those enrolled antenatally (51.9%) and during labor/delivery (54.5%), but significantly fewer postnatally enrolled infants achieved complete POC testing (36.4%, p = 0.01). No other demographic or clinical care variables were associated with complete POC testing.

### Efficiency

Of the total 853 POC tests run either at birth (n = 424) or at 6-weeks (n = 429), 803 (94.1%) were negative, 3 were positive (0.3%: 2 at birth, 1 at 6-weeks), and 47 (5.5%) did not have a valid result documented. Of the 806 with a valid result, 792 (98.2%) had documented notification of mother result. The mean turnaround time from sample collection to notification was 1.08 days, with 751 (94.8%) mothers notified on the same day as sample collection. ART was initiated in both infants diagnosed HIV-positive at birth, with turnaround times of 11 and 23 days between mother notification of result to ART initiation. The infant diagnosed as HIV-positive at 6-weeks did not initiate ART, due to maternal disengagement from care. Turnaround times and infant ages at key POC testing services by testing time point are displayed in Table 3.

**Table 3:** Efficiency of at birth and 6-week POC testing.

	Birth test	6-week test
	N = 424	N = 429
Mean Infant age at test (weeks)	1.07	6.29
N (%) with valid result^a^	388 (91.5%)	418 (97.4%)
Mean infant age at result availability (weeks)	1.07	6.43
N (%) mothers notified^b^	383 (98.7%)	409 (98.8%)
TAT: sample collection to mother notification (range)	0.48 days	1.65 days
	(0–61 days)	(0–139 days)
N (%) mothers notified on same day as sample collection^c^	365 (95.3%)	386 (95.5%)
Mean infant age at mother notification (weeks)	1.16	6.65
N (%) positive^c^	2 (0.5%)	1 (0.2%)

^a^Denominator is total number of tests

^b^Denominator is number of valid results

^c^Denominator is number of mothers notified ^c^Denominator is number of valid results.

#### POC implementation measures

In total, 47 POC samples did not produce a valid POC result: 36 at birth and 11 at 6-weeks. Machine error rates at birth and 6-weeks were 8.5% and 2.6%, respectively.

Missed opportunities for POC testing occurred when an infant had a documented PCR test at a given time point, indicating that they had presented for care, but did not receive a POC test at that time point. Of the 166 eligible infants who did not have an at birth POC sample collected, 48 (28.3%) presented for care and received a PCR test at birth. Of 195 eligible infants who did not receive a 6-week POC test, 150 (76.9%) presented for care and received a PCR test at 6-week. Thus, there were a total of 198 missed opportunities for POC testing (infant presented for testing but received standard PCR instead of POC testing). Documented reasons for these missed opportunities included cartridge stock out (n = 57), machine breakdown (n = 15), insufficient sample volume (n = 3), provider error (n = 12). Due to gaps in documentation, reasons for the remaining n = 111 missed opportunities are not explained.

In total, processing errors and missed opportunities resulted in 245 (23.3%) participants who presented for care not receiving a result, out of a total of 1,051 testing opportunities (853 samples collected + 198 missed opportunities).

Missed opportunities and processing errors accounted for 20.6% and 25.7% of all testing opportunities at GeneXpert and Alere m-PIMA sites, respectively, Table 4.

**Table 4:** Processing errors and missed opportunities.

	Alere m-PIMA	GeneXpert	Total
Total number of testing opportunities^a^	565	486	1,051
Valid result not available	41 (7.3%)	6 (1.2%)	47 (4.5%)
Missed opportunity: cartridge stock out	17 (3.0%)	40 (8.2%)	57 (5.4%)
Missed opportunity: machine breakdown	15 (2.7%)	0 (0%)	15 (1.4%)
Missed opportunity: insufficient sample	1 (0.2%)	2 (0.4%)	3 (0.3%)
Missed opportunity: provider error	9 (1.6%)	3 (0.6%)	12 (1.1%)
Missed opportunity: unknown reason	62 (11%)	49 (10.1%)	111 (10.6%)
Total	145 (25.7%)	100 (20.6%)	245 (23.3%)

^a^Total number of testing opportunities defined as the total number of samples collected (whether successfully processed or not) plus number of missed opportunities.

## Discussion

This evaluation demonstrates the potential for at birth POC testing to facilitate earlier infant HIV diagnosis and ART initiation. In total, 87% of infants had a POC sample collected prior to 12 weeks of age and most mothers were notified of their infant’s result on the same day as sample collection, representing an opportunity for ART initiation by the targeted 12 weeks of age. Nevertheless, the rate of complete testing was much lower, with only half of infants receiving both an at birth and a 6-week POC test. We allowed a generous window for birth (<4 weeks old) and 6-week (4–12 weeks old) testing. Kenyan [40] and international [33] guidelines more strictly define birth testing as occurring within 2 weeks of birth and the target for 6-week testing is by 2-months of age [41]. Using these cutoffs, fewer infants (41%) achieved the optimal sequence of “on-time” birth and “on-time” 6-week testing. Though only two infants were initiated on ART after a positive POC test, turnaround times between sample collection and ART initiation were very high, with neither infant initiated the same day as POC testing. Provider mistrust of accurate POC results–especially in very young infants–posed challenges to early ART initiation and resulted in providers sometimes opting to await the result of the PCR test prior to ART initiation [30, 42].

Since at birth testing only captures intrauterine HIV transmission, follow up testing remains essential to identify intrapartum and early breastfeeding transmission. Our data indicated that infants who received an at birth POC test were more likely to receive a second POC test by 12 weeks of age, compared to those who did not receive an at birth POC test. While a case-control study from South Africa indicated that high-risk infants who received at birth PCR testing were less likely to receive a repeat test than matched controls who did not receive an at birth test [19], our study and other similar studies from Eswatini, also did not support this finding [43]. Similarly, qualitative data suggests that mothers would find an initial negative result at birth encouraging and would motivate continued engagement in care [30]. Previous studies have demonstrated that maternal receipt of ART is associated with increased EID follow-up [44]; thus, caregivers of the most at-risk infants may be less likely to be retained in EID than the broader population of mother-infants pairs accessing PMTCT included in our evaluation. Still, 31% of infants did not receive a 6-week POC test—with 13% receiving no POC test—by 12 weeks of age.

While over half of all infants at 3 of the 4 sites received complete POC testing (54%-56.8%), one urban site evidenced much poorer rates of complete POC (35.9%). This site was a larger hospital in a high HIV prevalence area. Clinicians cited high workload, lack of space in ANC/MCH, and lack of systems for adequately tracking mother-infant pairs prior to 6-weeks as challenges to implementing at birth POC. Other studies have found that maintaining coverage over weekends and holidays [45], seeking delivery and postnatal care at different facilities [46], long turnaround times and low rates of linkage to care among infants diagnosed as HIV-positive [47] to be challenges for at birth PCR; while lack of consensus on who should test and where the testing should occur [43] and shortage of trained staff [30] have been cited as challenges to POC testing for EID.

Early enrollment (during ANC or labor/delivery) was the only other factor significantly associated with complete testing. This result was not surprising given that infants enrolled in the postpartum period would be ineligible for at birth testing, and therefore unable to receive complete POC testing, if enrolled >4 weeks of age. It does, however, highlight the importance of continued counseling throughout pregnancy to encourage mothers living with HIV who are engaged in care to seek timely at birth HIV testing for their infants [48]. It also highlights a need for interventions to target pregnant women living with HIV who are not engaged in PMTCT care, since these infants are not only at an increased risk of transmission [49] but also less likely to achieve complete infant testing [44]. Active tracking of mother-infant pairs throughout pregnancy and the early postpartum periods can improve early retention [4, 50, 51], helping to ensure timely infant HIV detection and diagnosis. Doing so may require developing systems to better track mother-infant pairs, including integration of PMTCT and MCH services [52, 53] and assignment of infant ID numbers at birth, rather than at the first MCH visit.

Errors, machine breakdown, cartridge stock outs, and other reasons resulted in 245 missed opportunities for POC results; which represented nearly a quarter of all testing opportunities. Alere m-PIMA sites experienced high rates of missed opportunities due to processing errors (especially at birth) and machine breakdown, while GeneX sites experienced high rates of missed opportunities due to cartridge stock-outs. These challenges have also been reported in other studies [22]. Implementers should ensure rigorous tracking of cartridge supply and expiration dates to minimize the occurrence of cartridge stock-out. Manufacturers should improve cartridge supply chains to expedite order delivery and accessibility globally and should work with implementers to ensure rapid service in the event of machine breakdown, to minimize the impact on clinical care. The GeneX error rate observed here (1.2%) was lower than both the error rate noted in a field evaluation study of GeneX, which employed several study staff to support implementation (4.8%) [18, 54] and the error rate noted in the WHO Prequalification Report (3%) [38, 55]. Likewise, the Alere m-PIMA error rate observed in this study (7.3%) was similar to the error rate in other evaluations of Alere m-PIMA (6–10%)(25–27) and to the rate reported in the WHO Prequalification report (5.8%) [39]. This indicates that with minimal extra training and support, clinical staff already employed within the hospital can successfully process POC samples using GeneX. The at birth POC error rate observed in our study (8.5%) was higher than at 6 weeks (2.6%), however comparable to that observed in other studies (4.8%- 11%) [17, 27, 43]. Higher error rates at birth could be a result of difficulty collecting adequate sample from infants.

Several limitations to our evaluation should be noted. Our total sample was relatively small; and we observed very few positive infants. Outcomes for HIV-positive infants–including timing of ART initiation and long term outcomes for HIV-positive infants–are an important aspect of evaluations for testing methods [31, 32]. While very low yield of positive infants’ results reflect the high coverage of antenatal ART and will likely continue to decrease, globally, as we strive towards elimination of mother-to-child transmission, it does prevent assessment of these critical impact data and forces us to rely on proxy measures such as timing of caregiver notification of results. Lack of retention and outcome data beyond the 6-week test also limits our ability to comment on long-term impact data. We enrolled women who presented for PMTCT and/or EID care; thus, this analysis only included those who were already engaged in HIV care. Rates of testing are likely lower among infants of women who are not engaged in care. Furthermore, reasons for nearly half of all missed opportunities were not documented. Missed opportunities were only documented as such if a standard of care PCR test was conducted at the same time-point. Given limitations in our data collection tools, we were unable to identify cases where a mother presented for care but received neither a POC nor PCR test. This hinders our ability to fully understand and describe the observed gaps in provision of POC testing. We also had limited maternal clinical information (viral load, default from own ART, ART adherence, disclosure status) that may influence complete testing. A national nurse’s strike restricted implementation during the first 5 months; to minimize the effect of the strike on outcomes, we excluded enrolled infants born during this period from analysis. Thus, while our study reflects implementation using existing hospital staff, it does not account for conditions that can disrupt routine service provision within hospitals. Lastly, our study included only four medium to high volume sites in Kenya, selected in part due to the presence of established research infrastructure, thus, limiting generalizability.

## Conclusion

These data indicate that at birth POC testing has the potential to streamline infant HIV diagnosis, expedite ART initiation, and after adequate training, can be successfully implemented by existing hospital staff. Mechanisms to engage mother-infant pairs who do not seek PMTCT/EID care, to improve mother-infant retention throughout the pregnancy and postpartum periods, and to ensure consistent cartridge supplies and timely machine repair are needed to realize the full benefits of at birth POC testing.
